# Metabolic and post-translational modifications in sepsis-associated immune dysfunction: a conceptual framework

**DOI:** 10.3389/fimmu.2026.1820993

**Published:** 2026-05-07

**Authors:** Mingze Xu, Ziye Zhang, Min Zhu, Sen Zhang, Juxin Deng, Zhaoyang Du, Zhenjie Wang, Hongchang Zhao, Zhaolei Qiu

**Affiliations:** 1 Department of Emergency Surgery, The First Affiliated Hospital of Bengbu Medical University, Bengbu, Anhui, China; 2 School of Life Science, Anhui Agricultural University, Hefei, Anhui, China; 3 Institute of Emergency and Critical Care Medicine, The First Affiliated Hospital of Bengbu Medical University, Bengbu, Anhui, China

**Keywords:** epigenetic regulation, immunosuppression, metabolic signaling, post-translational modification, sepsis

## Abstract

Sepsis is characterized by a progressive collapse of immune signal transduction, in which post-translational modifications (PTMs) act as critical execution layers that help shape the amplitude, duration, and reversibility of immune responses. Although often framed as a transition from cytokine storm to immune paralysis, the molecular logic governing this shift remains poorly defined. Growing evidence suggests that immune dysfunction in sepsis arises not from simple signal attenuation but from a loss of signaling competence, driven by coordinated changes in PTM networks, cellular metabolism, and chromatin structure. Here, we propose a metabolic–PTM temporal switch model as a conceptual framework in which immune signaling is rewired through context-dependent, PTM-associated configurations constrained by metabolic availability and chromatin accessibility. In early sepsis, permissive metabolic conditions and open chromatin may support fast, reversible PTMs—such as phosphorylation and scaffold-forming ubiquitination—that amplify innate immune signaling. As metabolic stress accumulates, a transition may occur in which ubiquitin linkage editing and increased deacetylation become more prominent and may contribute to the dismantling of signaling complexes and the restriction of transcriptional output. In late-stage sepsis, sustained metabolic exhaustion and chromatin condensation are associated with persistent PTMs, including histone lactylation, thereby potentially contributing to a low-plasticity immune state that becomes refractory to reactivation. Rather than implying a fixed temporal sequence, this framework is intended to describe representative PTM-associated patterns that may emerge across overlapping sepsis-related immune states. This framework may help explain why immune stimulation frequently fails in late sepsis: receptors and ligands may remain intact, yet signaling architecture and transcriptional competence can be substantially impaired. By identifying context-associated PTM patterns and signaling constraints, this model provides a conceptual basis for understanding context-dependent immune dysfunction and offers conceptual guidance for interpreting the variable outcomes of immune-targeted interventions in sepsis.

## 1 Introduction

Sepsis is a dysregulated immune response to infection that progresses from early hyperinflammation to late immunosuppression, leading to organ dysfunction and high mortality (1, 2). This process has traditionally been described as a biphasic transition. The initial phase involves cytokine-driven inflammation, which is followed by lymphocyte apoptosis, impaired antigen presentation, and increased vulnerability to secondary infections (3, 4). However, the driving mechanisms between these two phases remain incompletely understood. Clinically, hyperinflammatory and immunosuppressive features often coexist, and patients may exhibit features of both phases depending on pathogen burden, metabolic status, and cellular context (1, 5).

Recent studies suggest that post-translational modifications (PTMs) act as quick switches linking pathogen sensing to the cellular metabolic state (6). Through modifications such as phosphorylation, ubiquitination, acetylation, methylation, lactylation, PTMs regulate protein stability, localization, interaction, and transcriptional activity (7–9), enabling immune cells to adjust function without requiring new gene expression. These modifications do not act independently; instead, they function within layered regulatory networks where sequential and combined events may contribute to the shift from immune activation to suppression (10–12).

Initial studies showed that histone modifications contribute to endotoxin tolerance by regulating inflammatory gene expression, linking metabolism to chromatin remodeling (13). Subsequent studies revealed their distinct roles, including ubiquitination in inflammasome degradation (14), phosphorylation in MAPK activation (15, 16), and lactylation in linking lactate to transcriptional reprogramming (17–19). These findings suggest that PTMs influence both inflammatory amplification and resolution.

Despite extensive progress, major conceptual gaps remain. Metabolic signals that trigger changes in PTM patterns remain poorly understood. The functional hierarchy among co-occurring PTMs has yet to be clarified, and the specific role of lactylation in promoting immunosuppression—beyond its classification as a metabolic byproduct—remains uncertain. These unresolved questions emphasize major limitations of the traditional biphasic model, which primarily describes phenomena without fully explaining how metabolic decline, complex PTM regulation, and chromatin constraints interact during shifts in immune states.

**Figure 1:**
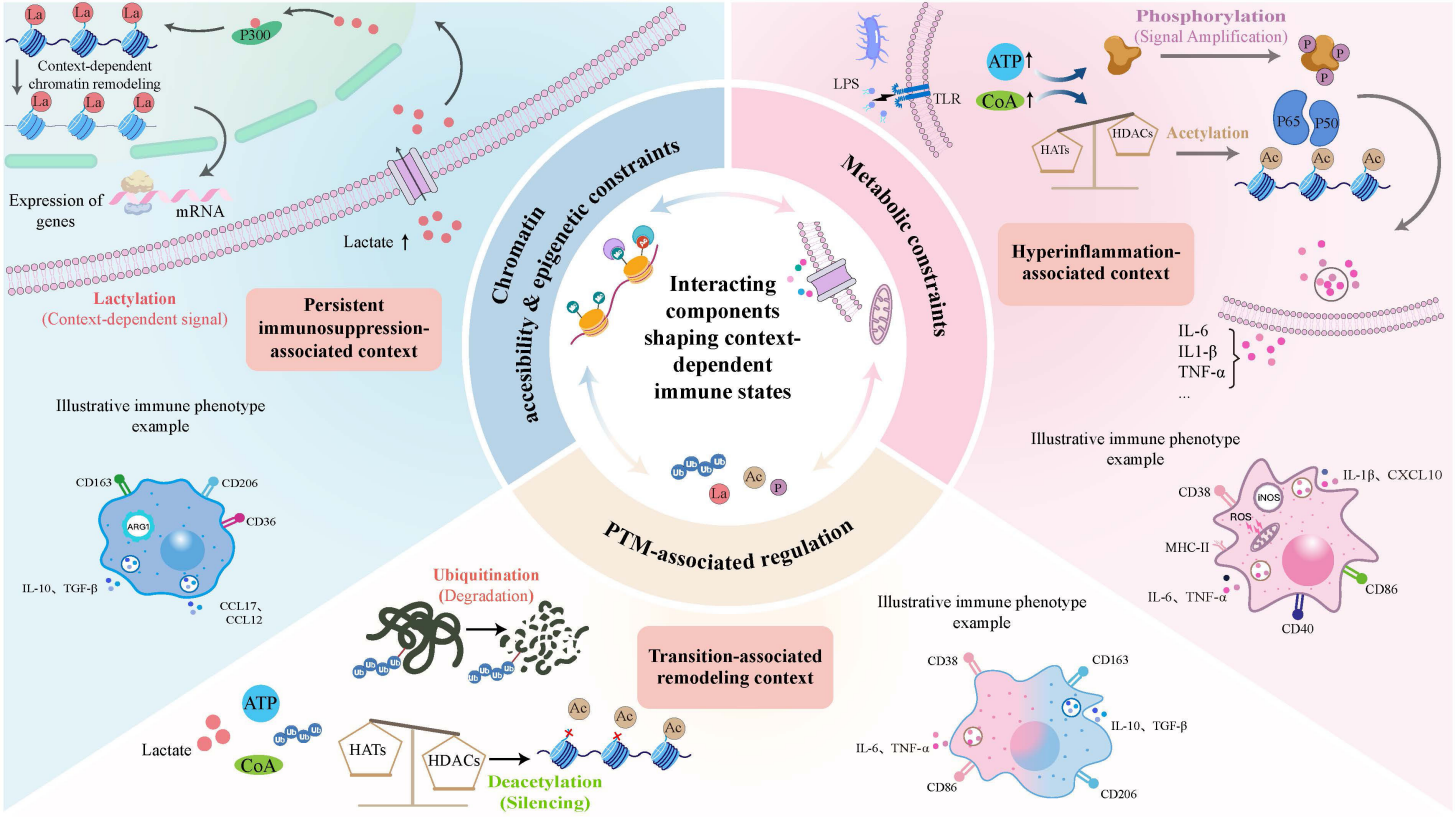
Conceptual framework of context-dependent interactions among metabolism, PTM-associated regulation, and chromatin accessibility in sepsis. This figure presents a conceptual and context-informed framework in which metabolism, PTM-associated regulation, and chromatin accessibility/epigenetic constraints are depicted as interacting components that shape immune states in sepsis. The depicted structure is intended to be illustrative rather than deterministic and does not imply a fixed or universally established sequence across all immune or non-immune compartments. Instead, the figure highlights three representative sepsis-associated immune contexts, including a hyperinflammation-associated context, a transition-associated remodeling context, and a persistent immunosuppression-associated context, in which different immune features, PTM-associated patterns, and chromatin-associated constraints may become more prominent. These context-associated configurations may influence whether immune signaling remains more permissive, more transitional, or more restrictively regulated. The macrophage phenotypes are provided as an illustrative myeloid example and are not intended to represent a universal trajectory across sepsis contexts.

To address these conceptual gaps, we propose a Metabolic–PTM Temporal Switch model (**Figure 1**). In this model, the availability of specific substrates and chromatin accessibility within each immune context may help shape the relative prominence and interaction of PTM-associated patterns and guide changes in the immune state. This framework may also help identify enzymes and metabolic requirements that remain pharmacologically targetable during specific immune states in sepsis.

## 2 PTMs operate within a multi-layered regulatory architecture: metabolic gating and epigenetic silencing

**Figure 2:**
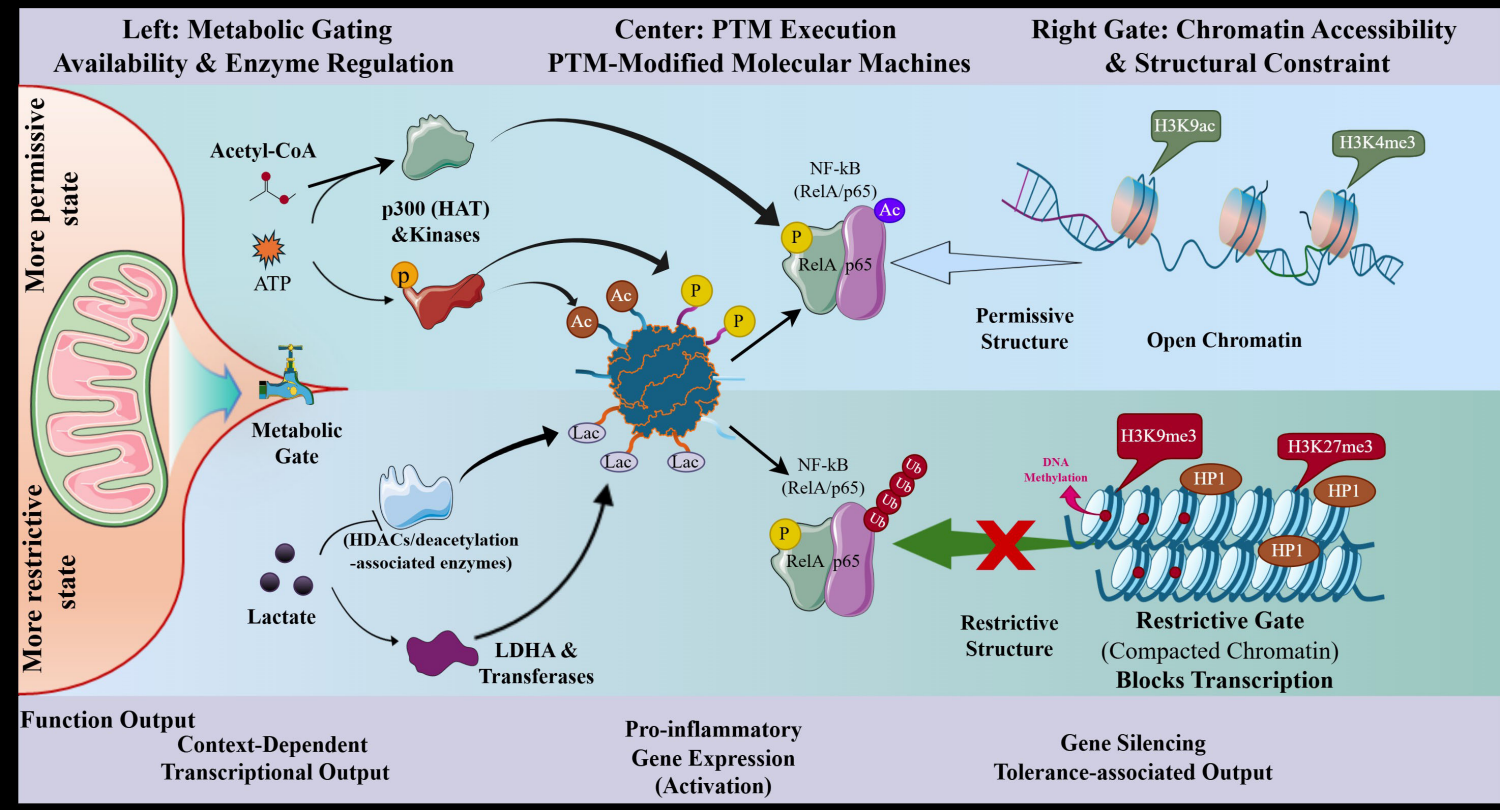
PTMs operate within two major constraints: metabolism and chromatin accessibility. The figure illustrates the functional coupling between metabolic flux, histone PTMs, and DNA methylation, highlighting them as interconnected regulatory features rather than isolated processes. PTMs are shown here as components of immune signaling whose effects are constrained by two major regulatory contexts. The metabolic component (left) influences whether PTMs are chemically feasible by providing substrates such as ATP, acetyl-CoA, NAD^+^, and lactate, while also shaping enzyme activity. Under more permissive metabolic conditions, this may favor PTMs such as phosphorylation and acetylation, whereas under more restrictive metabolic conditions it may favor lactylation and deacetylation-associated restrictive states. The PTM component (center) integrates these inputs to modify signaling proteins and transcriptional regulators such as NF-κB/RelA through phosphorylation, acetylation, ubiquitination, and lactylation. The chromatin component (right) shapes whether these PTM-associated signals can be translated into transcriptional outputs. Open chromatin states (e.g., H3K9ac and H3K4me3) are associated with gene accessibility, whereas restrictive chromatin states (e.g., H3K9me3, H3K27me3 with HP1 and DNA methylation) are associated with transcriptional silencing. This framework is intended as a conceptual and context-informed schematic and does not imply a fixed or universally conserved temporal sequence *in vivo*. It may help explain why immune activation can fail in more restrictive sepsis-associated immune states.

Post-translational modifications (PTMs) do not function in isolation but operate within a regulatory framework defined by metabolic availability and chromatin organization (20). These two upper-tier layers help define the biochemical and transcriptional boundaries within which PTMs can act. Metabolic cofactors influence which modifications are chemically feasible, whereas chromatin accessibility shapes whether those modifications can be converted to transcriptional consequences. Within this dual-constraint architecture, PTMs can be viewed not as independent contributors to immune behavior, but rather as downstream effectors that may implement outcomes encoded by metabolic flux and epigenetic structure (**Figure 2**).

### 2.1 Metabolic cofactors shape PTM feasibility

In early sepsis, the body may establish a glycolysis-dominant metabolic environment. This increases the levels of ATP and acetyl-CoA, which supply phosphate and acetyl groups for efficient phosphorylation and acetylation. At the same time, this process quickly triggers inflammation by turning on inflammatory genes such as TNF, IL6, and CXCL8 (21–23). As disease progresses, mitochondrial dysfunction and redox imbalance reduce NAD^+^ availability, increasing the influence of deacetylases and limiting acetylation-dependent activation (24). When lactate accumulates, it may also contribute to lactylation-associated changes through lactyl-CoA, potentially participating in immune suppression in later sepsis-associated immune states (25–27). Through these shifts, metabolic deterioration may contribute to reshaping the PTM patterns, progressively shifting the relative prominence of activating to suppressive modifications. Metabolism may thus function as a biochemical axis that influences the feasibility of specific PTM reactions within a given immune context in sepsis. Metabolic signals do not merely control PTM feasibility but may also influence chromatin-state transitions. For example, NAD^+^ depletion and broader metabolic stress may shift the acetylation–deacetylation state toward deacetylation-associated chromatin repression, thereby enforcing transcriptional boundaries that precede more persistent immune dysfunction (28).

### 2.2 Dual epigenetic layers: histone modifications and DNA methylation

Whereas metabolism determines PTM feasibility, chromatin accessibility determines PTM effectiveness. Sepsis-induced tolerance is characterized by the targeted deposition of repressive histone marks such as H3K9me2/3 and the recruitment of HP1 and DNMTs, which compact chromatin at promoters of key cytokines including *TNF*, *IL6*, and *CXCL* family genes (29–31). These modifications do not trigger global transcriptional shutdown but instead generate a selective silencing map that renders inflammatory loci inaccessible even in the presence of intact upstream signaling (32). Under such conditions, activating PTMs may continue to accumulate on transcription factors or cofactors but cannot restore transcriptional output (33, 34). Rather than being an ancillary event, DNA methylation may represent a parallel and durable epigenetic layer that operates in synergy with histone PTMs to encode the ‘long-term memory’ of the septic insult (35–37). Beyond histone-based chromatin remodeling, promoter or enhancer methylation at inflammatory and antigen-presentation genes can contribute to persistent transcriptional silencing and stabilization of tolerant immune states. Early work in endotoxin tolerance showed that TNF transcriptional silencing is associated with coordinated H3K9 methylation, HP1 recruitment, Dnmt3a/b binding, and promoter CpG hypermethylation, supporting functional coupling between histone and DNA methylation during inflammatory gene repression (38).

More recent studies further suggest that this process is metabolically linked: infection or LPS induced immune tolerance is accompanied by TCA-associated metabolic rewiring and DNA hypermethylation of innate immune genes, whereas metabolic interventions can mitigate hypermethylation and partially restore immune responsiveness (39). DNA methylation is also metabolically coupled through one-carbon metabolism and related methyl-donor availability for DNMT activity, while TCA-derived metabolites can influence TET-dependent demethylation and broader epigenetic plasticity. These observations also align with broader work on innate immune memory showing that tolerance and trained immunity are both accompanied by durable but distinct epigenetic remodeling programs (40, 41). In this context, DNA methylation should be viewed not as separate from the metabolic–PTM framework proposed here, but as a complementary and more persistent epigenetic layer that may help stabilize long-term immune dysfunction in sepsis.

### 2.3 Combined constraint logic

Metabolic gating and chromatin accessibility jointly define the PTM operating space (42, 43). Biochemically permissive conditions and accessible chromatin allow activating PTMs to spread inflammatory transcription (44, 45), whereas metabolic decline shifts PTMs toward suppressive forms whose impact depends on whether target loci remain open (43, 46, 47). Once heterochromatin is established, activating PTMs become transcriptionally inactive (37, 48), and suppressive PTMs deposited during this period help stabilize tolerant states (49, 50). This combined control system creates a specific regulatory setup for each immune context. It influences not only which PTMs happen but also whether they affect function.

### 2.4 Chromatin remodeling as a basis for long-term immune memory

Beyond its role in acute tolerance, chromatin remodeling imprints durable structural states that extend well beyond the inflammatory period (51, 52). Supporting this concept, ATAC-seq and ChIP-seq analyses of monocytes from septic patients demonstrate a sustained redistribution of activating marks (H3K4me3, H3K9ac) and repressive marks (H3K27me3, H3K9me2) at loci involved in antigen presentation, interferon signaling, and cytokine synthesis (53). These epigenetic alterations predispose myeloid cells to sustained hypo-responsiveness and limit their capacity to re-engage inflammatory programs, even after upstream signaling has normalized. In this case, PTMs work within a chromatin structure that has already been established. This affects how long and how strong the immune response will be.

### 2.5 Summary of foundational logic

Together, metabolic state and chromatin organization define the biochemical and transcriptional boundaries within which PTMs shape immune behavior (54–56). Metabolic cofactors determine which PTMs can be added (57, 58). Chromatin accessibility decides if these changes affect gene activity. The interaction between these two layers influences whether the immune system is activated, restrained, or suppressed. PTMs act as molecular tools that help turn complex metabolic and chromatin signals into changing immune responses (59). This idea of dual constraints is a key concept in understanding sepsis immunobiology (60, 61). From a translational standpoint, this implies that effective PTM-targeted intervention may require combinatorial strategies—such as metabolic support or chromatin-modulating agents—to reopen transcriptional competence before direct PTM modulation.

## 3 A conceptual framework of context-dependent ptm patterns in sepsis-associated immune states

Metabolic and chromatin factors set the limits for what regulation is possible. Within these constraints, post-translational changes may occur in a broadly ordered manner rather than all at once. These PTM-associated patterns may help organize immune states ranging from acute activation to signal restriction and more persistent silencing. This section outlines how specific classes of post-translational modifications—phosphorylation, ubiquitination, acetylation, and lactylation—may contribute to distinct immune states in sepsis.

### 3.1 Early activation: phosphorylation acts as a fast signal booster

Phosphorylation is among the earliest and most rapidly induced PTMs following pattern-recognition receptor engagement (62–64). Kinase cascades such as MAPK, JNK, TBK1, and p38 convert receptor stimulation into immediate functional changes—enhancing adaptor assembly, initiating NF-κB and IRF activation, and priming inflammasome components (65–67). Because phosphorylation requires no new protein synthesis and is easily reversible, it operates as a high-speed amplification layer.

A key feature of phosphorylation is its early regulatory role within PTM-associated signaling networks (68). Phosphorylation changes the shape of a substrate, which can either reveal or hide lysine residues for later addition of ubiquitin (69, 70). It controls whether scaffold assemblies are kept stable or broken down (71–73). It may also facilitate later acetylation-associated activation of transcription factors (74). Thus, phosphorylation not only drives the initiation of inflammation but also configures substrates for subsequent PTM events. Because phosphorylation remains highly reversible and enzymatically tractable, this early PTM layer makes kinase signaling an attractive early-stage intervention point to restrain hyperinflammation without enforcing long-term immunosuppression.

### 3.2 Transition-associated context: ubiquitination-mediated proteostatic resolution

As inflammatory signaling progresses, ubiquitination may become a prominent PTM layer that helps shift the system from propagation to restraint (75, 76). Early activation relies on K63- and M1-linked chains, which stabilize receptor complexes (MyD88, TRAF6, NLRP3), maintain MAPK/NF-κB competence, and act as scaffold platforms that prolong phosphorylation-driven signaling (77–79).

A key inflection point occurs when these scaffolding chains are replaced by degradative K48-linked ubiquitination, driven by inducible E3 ligases whose activity rises under metabolic and redox stress (80, 81). Cbl-b (upregulated by sustained TLR/MAPK signaling and Ca²^+^ influx) (82, 83), RNF125 (induced by type I IFNs and hypoxia) (84), and FBXO3 (activated by ROS and ER stress) together may promote a directed K63→K48 switch, thereby favoring proteasomal degradation of signaling complexes (85–87).

DUBs modulate but do not reverse this transition (88). A20, oxidatively inactivated at its zinc-finger domains, loses K63-editing capacity (89); CYLD, dependent on ATP/NAD^+^, becomes limited as energy declines; USP7 preferentially promotes degradative states under hypoxia or redox imbalance (90). Thus, metabolic deterioration may bias DUB activity toward reinforcing the shift toward degradation (91).

The emergence of K48-linked ubiquitination is proposed to represent a functional shift toward proteasomal turnover, beyond which signaling components are progressively lost (92–94). Unlike reversible modifications such as phosphorylation or acetylation, K48-linked degradation can more durably disrupt signaling architecture (95). This may help explain why immune pathways in late sepsis remain inactive despite the presence of intact receptors and persistent ligands (96).

Accordingly, ubiquitination may occupy a central regulatory position in shaping whether immune signaling is sustained or progressively terminated. Ubiquitination may connect early immune activation to later resolution by shifting from scaffold-forming ubiquitin chains to degradation signals. This process may also contribute to chromatin and transcription-associated states that become more restrictive later in sepsis. The linkage-specific switch in ubiquitination indicates that selectively targeting E3 ligases or deubiquitinases can either restore signaling function or speed up resolution, depending on the context of the disease. This makes ubiquitin-editing enzymes promising yet time-sensitive therapeutic targets.

### 3.3 Acetylation/deacetylation: a transcriptional filter in transition-associated states

After phosphorylation and changes to the scaffold through ubiquitin, acetylation and deacetylation influence whether signals from those steps lead to changes in gene activity (97). Acetylation of histones and transcription factors stabilizes promoter occupancy and enhances transcriptional output, which allows signals from earlier steps to pass through a point that permits expression to occur (98–100).

As deacetylation becomes more prominent, this regulatory checkpoint progressively tightens. Deacetylases such as HDAC1, HDAC2, and SIRT1 reverse activating acetyl marks, thereby narrowing the effective transcriptional window and constraining inducible gene expression, even when phosphorylation and scaffold-forming ubiquitination remain active (101, 102). In this context, the balance between acetylation and deacetylation critically shapes whether upstream PTMs can be translated into transcriptional output.

Within PTM-associated regulation, this layer functions as an intermediate gating mechanism, integrating upstream signaling intensity with downstream transcriptional capacity. This control point in gene activity focuses on enzymes called acetyltransferases and deacetylases. These enzymes can be targeted in two ways: by blocking or activating them. But any treatment needs to match the body’s metabolic state to avoid turning off gene activity too soon.

This positions acetyltransferases and deacetylases as bidirectional therapeutic levers, whose modulation must be aligned with disease context to avoid premature chromatin closure or ineffective immune stimulation.

### 3.4 Lactylation as a context-dependent metabolic–epigenetic signal in sepsis

Lactylation may become more evident in later sepsis-associated immune states, particularly under conditions of metabolic collapse, chromatin condensation, and reduced immune flexibility (103, 104). Rather than being viewed as a universally terminal suppressive PTM, lactylation is better interpreted as a context-dependent metabolic–epigenetic mark whose functional consequences may differ across immune activation, repair, tolerance, and trained-immunity settings. Under excess glycolysis and mitochondrial failure, pyruvate is diverted to lactate, generating lactyl-CoA, which fuels histone and non-histone lactylation (105–107). The acetyltransferase p300, capable of incorporating lactyl groups when lactyl-CoA is abundant, links metabolic overflow to epigenetic reprogramming (108), which may partly explain why lactylation becomes more evident during energy exhaustion rather than early inflammation.

A defining property is its biochemical persistence. Unlike acetylation, which is rapidly removed by HDACs or sirtuins (109–111), lactylation shows slow turnover, and no mammalian delactylase has yet been conclusively validated. This relative irreversibility suggests that lactylation may override earlier activating PTMs and stabilize transcriptional repression even after upstream signals partially normalize.

Histone lactylation—especially H3K18la—has been associated with reparative or suppressive transcriptional programs at loci that become unresponsive in late sepsis, acting as a metabolic-epigenetic rheostat that, depending on the immunological context, may either support tissue repair or contribute to the maintenance of tolerant states (112, 113). Non-histone lactylation modifies transcription factors, RNA-binding proteins, and metabolic enzymes (18, 106, 107, 112). For example, lactylation of glycolytic enzymes may increase metabolic dependency, while changes to transcriptional regulators can alter DNA binding or interaction networks, reinforcing the shift toward immune paralysis. Proteomics indicates that lactylation preferentially targets nodes central to immune–metabolic crosstalk (114, 115).

Collectively, these features suggest that lactylation may contribute to stabilizing some more persistent immunosuppression-associated states within the proposed PTM framework. Instead of causing changes like phosphorylation or ubiquitination, it may help stabilize specific gene programs, which can manifest as either persistent suppression or, in other settings, trained innate immunity, by lowering signaling flexibility and embedding aspects of the cell’s metabolic history in chromatin-associated programs. Although direct delactylation strategies are currently unavailable, the lactate–lactylation axis nevertheless suggests a potentially metabolically addressable vulnerability in late sepsis. At the same time, its effects are likely to be context-dependent and may differ across immune activation, tolerance, and trained-immunity settings, as recent studies suggest that lactate-driven histone lactylation can also support innate immune memory and trained immunity programs beyond immunosuppressive phenotypes (116, 117).

### 3.5 Conceptual summary of PTM roles across sepsis-associated immune states

Across sepsis-associated immune states, PTMs may form interacting and partially overlapping regulatory patterns rather than a collection of isolated pathways. Phosphorylation can support rapid inflammatory signaling (118, 119). Ubiquitination may help determine whether these signals are sustained or progressively terminated (120–122). Acetylation and deacetylation influence whether upstream signals are translated into gene activity (123–125), and lactylation may become more prominent in some persistent reprogrammed states (126, 127). Each PTM layer can influence the context in which other PTM events become more prominent, although the relative timing and dominance of these interactions are likely to vary across cell types, tissues, and disease.

These interacting PTM patterns may contribute to directionality, persistence, and context specificity in immune transitions: activation may be associated with phosphorylation-driven priming (128, 129), resolution may involve ubiquitin-mediated proteostasis and deacetylation (130, 131), and more persistent immunosuppressive states may show lactylation-associated stabilization (132). Immune fate is therefore likely encoded not by any single PTM, but by the structure and context of their interactions, with PTMs functioning as proximate molecular regulators that may help shape real-time transitions between physiological states.

## 4 A conceptual framework for PTM-associated reprogramming in sepsis

Sepsis progression reflects a systems-level reorganization of immune signaling rather than a linear rise and fall in inflammatory strength (133, 134). The metabolic and chromatin constraints outlined in Section 2 define the biochemical and structural boundaries within which PTMs operate, but the coordination among PTMs may also contribute to the emergence of distinct immunological states. Here, we propose a conceptual framework to explain how immune changes may arise through the interaction of metabolism, chromatin accessibility, and PTM-associated regulation. This framework views sepsis as a dynamic system in which the immune system may shift as PTM patterns change over time.

### 4.1 Conceptual framework: the metabolic–PTM temporal switch

The Metabolic–PTM Temporal Switch is proposed as a conceptual framework for understanding how immune cells may change over time. It provides a conceptual description of how immune cells may transition from activation to restraint and, in some contexts, to more persistent suppression. Rather than treating hyperinflammation and immunosuppression as opposing conditions, this model presents sepsis as a constraint-driven process in which metabolism, chromatin organization, and PTM-associated regulation interact. Metabolic cofactors and chromatin organization may influence which post-translational modifications (PTMs) occur, and these PTMs in turn may help shape directional changes in immune responses (113, 135–137). Metabolites like ATP, acetyl-CoA, NAD^+^, and lactate may influence whether particular PTMs are chemically favored under specific conditions. At the same time, chromatin accessibility affects how PTMs influence gene activity. In addition to histone-centered chromatin remodeling, durable DNA methylation programs should also be considered a parallel and metabolically coupled epigenetic layer that may help stabilize longer-term immune reprogramming in sepsis. Together, these factors may influence PTM patterns as sepsis develops. At present, this framework is most directly supported by evidence from myeloid-cell reprogramming and endotoxin-tolerance–like settings, and its applicability to other immune or non-immune compartments in sepsis remains to be established.

Much of the mechanistic logic discussed here is derived from endotoxin-tolerance models and *in vitro* myeloid systems, whereas patient-derived sepsis datasets mainly provide disease relevance and correlative support rather than full temporal mechanistic resolution. Importantly, the framework proposed here is not intended to imply a fixed or universally conserved temporal sequence of PTMs *in vivo*. Rather, it is a conceptual and context-dependent model describing PTM-associated configurations that may become more prominent across different sepsis-related immune states. These states may overlap substantially, and their relative PTM composition is likely shaped not only by metabolism but also by receptor signaling, cell identity, tissue context, and compartment-specific constraints. Metabolism should therefore be viewed as a co-regulatory axis that operates in bidirectional coupling with receptor signaling and cell identity, rather than as the sole upstream determinant of PTM ordering.

Within this shifting boundary, PTM-associated patterns may operate in a broadly ordered manner. In more inflammatory contexts, high energy use and open chromatin may favor phosphorylation and scaffold-forming ubiquitination, thereby supporting rapid signaling responses (138). As resources decline and chromatin compacts, ubiquitin linkage editing and deacetylation-associated states may become more prominent, dismantling signaling complexes and restricting transcription (139–141). Under conditions of marked metabolic and structural inflexibility, lactylation and other more persistent PTM-associated programs may help consolidate a tolerant state (106, 142). In this sense, individual PTM layers may not only execute proximal functions but also reconfigure substrate states for subsequent regulation.

A central feature of the model is PTM-associated temporal memory. Early PTMs may arise quickly and remain relatively reversible. Later, more persistent chromatin-associated changes may retain aspects of the cell’s metabolic and signaling history (143). These enduring marks—not receptor desensitization alone—may underlie long-lasting impairments in antigen presentation, responsiveness, and exhaustion-like phenotypes in late sepsis (144). PTMs may therefore function as molecular integrators of transient metabolic changes into longer-term immune states (145).

By conceptualizing sepsis as a process of PTM-associated immune reprogramming constrained by metabolism and chromatin, the model reframes immune dysfunction as an emergent property of a multi-layered regulatory system rather than isolated pathway failure. This provides the conceptual foundation for the representative PTM patterns discussed in subsequent sections. From this perspective, treatment efficacy may depend less on signal intensity alone and more on whether intervention is matched to the dominant PTM-associated constraints present at a given disease context.

Importantly, this framework should not be interpreted as exclusive to sepsis. Related forms of critical illness, including severe burns and trauma, also show evidence of immunometabolic and epigenetic reprogramming, including glycolytic stress responses, durable DNA methylation changes, and histone-based inflammatory rewiring. In this sense, the Metabolic–PTM Temporal Switch may capture broader principles of critical illness–associated immune adaptation. However, sepsis likely differs in the intensity and persistence of these processes because infection introduces sustained or recurrent PAMP-driven signaling on top of sterile DAMP-associated injury responses, which may favor more pronounced tolerance-associated immune dysfunction.

### 4.2 High-plasticity pro-inflammatory configurations

In the early hyperinflammatory context, metabolic and chromatin conditions may remain permissive enough to support fast-acting PTMs that initiate and amplify innate immune signaling (55, 136, 146). Within this environment, phosphorylation and scaffold-forming ubiquitination may act as prominent regulatory layers that convert pathogen-recognition events into immediate functional outputs. Phosphorylation rapidly activates MAPK, TBK1, and related kinase pathways, allowing transcription factors and inflammasome-associated components to become engaged, without requiring new protein synthesis (147–149). In parallel, K63-linked ubiquitination stabilizes receptor-proximal assemblies—including MyD88, TRAF6, and NLRP3—thereby sustaining signaling competence and permitting efficient propagation of inflammatory cues (150, 151).

Acetylation contributes to this context not by dictating immune trajectory but by preventing early transcriptional bottlenecks. Through the modification of histones and key transcription factors (152), acetylation maintains an accessible chromatin environment and supports promoter occupancy once kinase signaling initiates gene expression. Its role in this setting is therefore permissive rather than directive: acetylation enables the transcriptional readout of upstream PTM activity but does not by itself determine the fate of the response.

Collectively, this context is characterized by relatively high-plasticity PTMs that prioritize speed, reversibility, and signal amplification. Phosphorylation provides an immediate catalytic trigger (153, 154), K63-linked ubiquitination supplies structural reinforcement for sustained signaling, and acetylation supports transcriptional throughput (155, 156). As these processes intensify, they may place growing demands on metabolism and protein homeostasis, creating conditions that could favor a subsequent shift toward more suppressive PTM-associated programs such as K48-linked ubiquitination and deacetylation. Thus, this early context may represent a state in which immune signaling remains highly responsive and dynamically regulated.

### 4.3 Signaling restriction and remodeling configurations

As early hyperinflammation subsides, PTM regulation may enter a transition-associated remodeling context in which the balance shifts from sustaining signaling toward progressively dismantling it. Under tightening metabolic and chromatin constraints, PTMs may assume different roles: amplification gives way to proteostatic turnover, and transcriptional permissiveness shifts toward selective silencing. This context may represent an important inflection point at which immune programming begins to change direction.

A defining event may be the functional inversion of ubiquitination. Cbl-b, RNF125, and FBXO3 can mediate the replacement of scaffold-forming K63-linked chains on receptor and inflammasome complexes with K48-linked ubiquitination (157–159). This process directs key adaptors toward proteasomal degradation, eliminating the structural substrates necessary for MAPK and NF-κB activation (160–163). DUBs such as A20 and CYLD edit—but do not reverse—this shift, thereby influencing the precise timing of scaffold collapse (164, 165). Through this linkage editing, ubiquitination may become an important mechanism contributing to signal termination (166).

In parallel, acetylation may shift toward more prominent deacetylation-associated states. Sirtuins and HDACs remove activating histone and transcription factor acetyl marks, narrowing promoter accessibility and reducing the range of inducible gene programs (167–170). This creates a transcriptional bottleneck that upstream PTMs may no longer fully overcome.

Together, proteasomal decay and transcriptional narrowing create a bifurcated regulatory architecture: signaling pathways lose structural continuity, while transcriptional machinery loses access to inflammatory loci. Rather than global paralysis, immune cells may undergo selective pathway pruning, with antimicrobial and antigen-presentation functions declining while reparative circuits remain relatively preserved—laying the foundation for tolerant or exhaustion-like states.

Thus, this transition-associated context may mark a shift from reversible activation toward more directionally constrained immune regulation, while establishing conditions in which persistent deacetylation and potentially stabilizing lactylation-associated programs become more evident.

### 4.4 Low-plasticity and stabilization configurations

In late sepsis-associated immune dysfunction, the system may shift from a still regulatable tolerant state toward a more fixed, low-plasticity immune configuration in which inflammatory programs become difficult to reactivate even with renewed stimulation. Unlike transition-associated states, where metabolic and chromatin cues may still redirect signaling, this context may approach a more constrained state defined by rigid signaling architecture, compact chromatin, and a restricted PTM repertoire (171). Here, PTMs may function less as flexible controllers and more as marks that help maintain transcriptional repression (144).

In this setting, the PTM landscape may narrow toward modifications associated with quiescence. Persistent deacetylation may stabilize closed chromatin at inflammatory loci (23), while lactylation may accumulate in association with reparative or anti-inflammatory programs (172–175). These marks may act as relatively self-sustaining signatures, preserving repression independently of external signals. Their persistence beyond partial metabolic recovery suggests a PTM-based molecular memory that embeds prior stress into more durable transcriptional states (176, 177).

Simultaneously, proteostatic mechanisms may dismantle signaling capacity. Continued K48-linked ubiquitination may prevent reassembly of inflammasome and PRR scaffolds (178, 179), and lysosomal degradation may deplete key adaptors required for NF-κB and MAPK activation (180). Adaptive immune cells may acquire additional constraints via inhibitory phosphorylation and stabilized checkpoints, further limiting effector potential (181, 182). As a result, signaling networks may become effectively severed: although receptors remain intact, downstream intermediates are missing, misplaced, or inactive. This disruption may prevent reactivation without substantial protein resynthesis and epigenetic reprogramming. Collectively, these processes suggest that late immunosuppression may reflect not merely passive decline, but a more persistent state emerging from interacting PTM-associated, metabolic, and chromatin constraints. This state may be stabilized by PTMs and epigenetic programs that become more prominent in late sepsis, thereby helping explain the persistence of immune dysfunction after resolution of the initial inflammatory triggers.

This context-dependent pattern reflects changes in metabolism and chromatin, while also illustrating how PTMs may interact through partial ordering, mutual exclusivity, and overwrite-like relationships. Together, these features may help explain why certain PTM-associated patterns become more prominent in different sepsis-related immune states.

**Figure 3:**
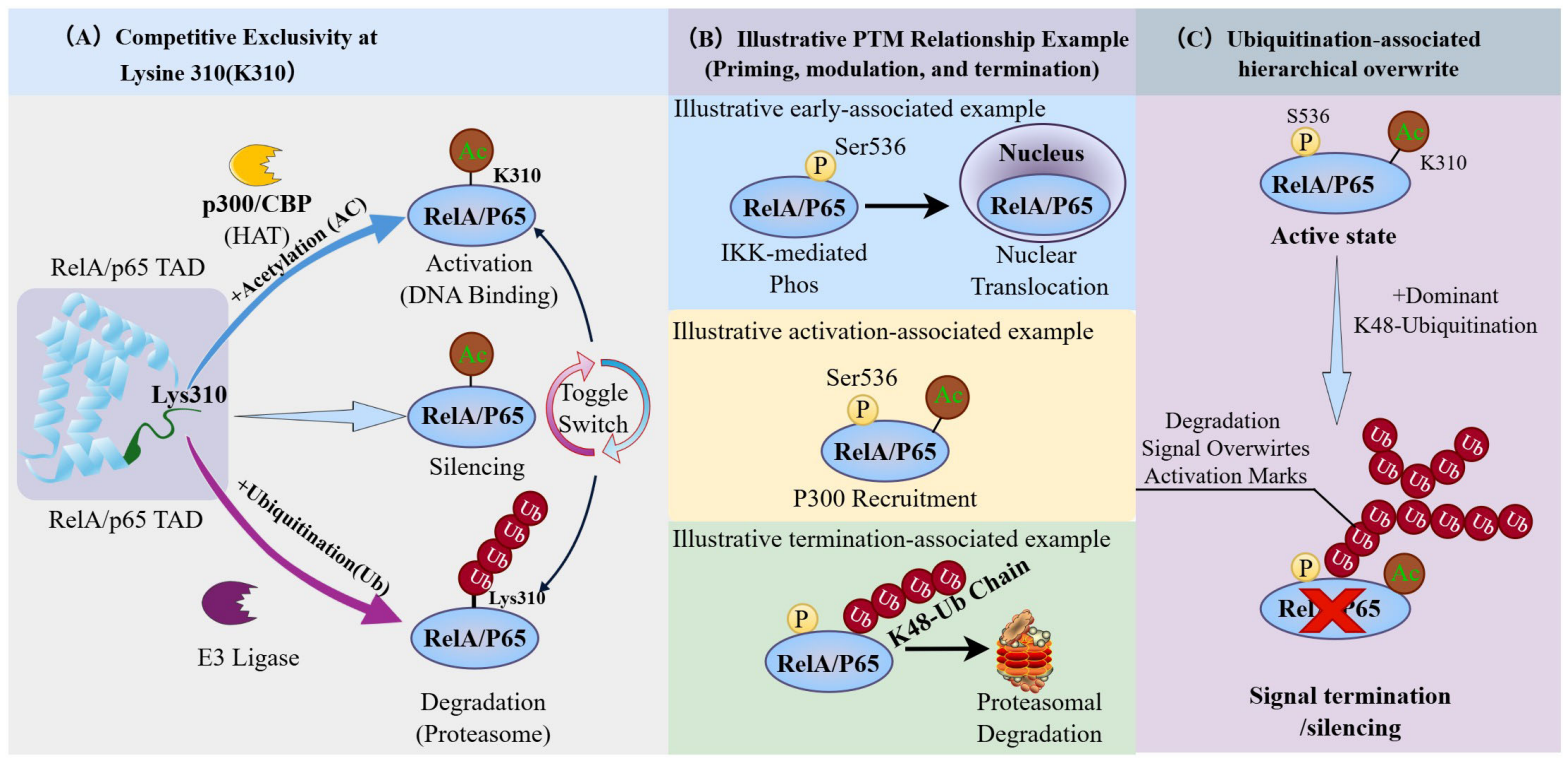
Principles guiding PTM interactions and signaling outcomes. This figure is intended as an illustrative and context-dependent schematic and does not imply a fixed or universally conserved temporal sequence *in vivo*. **(A)** PTMs may compete at the same lysine site, shown by RelA/p65 Lys310. Acetylation at this site is generally associated with enhanced transcriptional activity, whereas ubiquitination promotes proteasomal turnover. Because these modifications are mutually exclusive at the same residue, they may lead to distinct functional outcomes. **(B)** In some signaling contexts, PTMs may occur in a broadly or partially ordered manner. In NF-κB signaling, phosphorylation (e.g., Ser536) may facilitate nuclear translocation, followed by acetylation that supports transcriptional activity. Subsequent K48-linked ubiquitination may promote protein degradation and signal termination. **(C)** Some PTMs may functionally override others. For example, K48-linked ubiquitination can terminate signaling despite the presence of activating marks. Depending on context, more persistent chromatin-associated programs, including histone lactylation, may also contribute to more restrictive signaling states. Together, these examples illustrate how PTM interactions may influence whether signaling remains reversible or becomes more durably suppressed.

These observations set the stage for the mechanistic analysis presented in Section 5. The immune state transitions described in Section 4 may arise from PTM interaction rules, including partial ordering, competitive exclusivity, and hierarchical overwrite, which together provide a conceptual basis for understanding the switch-like behavior of immune cells. These crosstalk principles are discussed in the following section (**Figure 3**).

## 5 General principles of PTM crosstalk relevant to sepsis

Following the conceptual framework proposed, here we discuss general principles of PTM crosstalk that may help explain temporal switching in sepsis-relevant contexts. Rather than occurring randomly, PTMs may follow interaction principles—including sequential ordering, competitive exclusivity, and hierarchical overwrite—that may influence signal directionality, reversibility, and fate during sepsis progression (**Figure 3**).

Post-translational modifications (PTMs) do not operate as isolated biochemical events (11, 183). Instead, they form an integrated regulatory network in which different modifications influence, enable, or oppose one another to determine immune signaling outcomes (184). These interactions arise from shared substrates, overlapping modification sites, and multi-layered control of protein stability and activity (183, 185). In sepsis, these interactions may organize PTM patterns into coordinated control states. These states affect how strong, how long, and what type of immune responses happen (172, 186, 187).

This section outlines four core principles of PTM interaction—sequential ordering, competitive exclusivity, combinatorial encoding, and multi-PTM control of shared targets—which together create a higher-order regulatory architecture guiding immune fate.

### 5.1 Sequential PTMs and directional immune signaling

In some immune signaling contexts, sequential PTM waves may represent an important organizational principle (188). Rather than isolated events, PTMs may occur in partially ordered cycles, where each modification alters substrate conformation, complex assembly, or localization, thereby creating the prerequisites for the next (189). Such partially ordered progression may contribute to directionality—from initiation, to amplification, to termination—allowing immune responses to unfold as structured programs (190).

The NLRP3 inflammasome exemplifies this logic (191, 192). Early K63-linked ubiquitination stabilizes NLRP3 scaffolds and licenses initial assembly (193, 194). Subsequent phosphorylation enhances enzymatic competence and oligomerization, amplifying IL-1β maturation (159, 195, 196). Later, K48-linked ubiquitination targets the same components for proteasomal degradation, dismantling the inflammasome (87, 192, 197, 198). These sequential waves suggest that NLRP3 activation is both rapidly inducible and intrinsically self-limiting (85, 199).

A similar pattern governs TLR and IL-1R signaling (188). Phosphorylation of IRAKs creates docking sites for E3 ligases like TRAF6, whose K63-linked ubiquitin chains stabilize complexes required for MAPK and NF-κB activation (200–202). As signaling continues, DUBs and degradative ligases are recruited in a defined order to collapse the scaffold and impose termination checkpoints (203).

Thus, sequential PTMs may function as directional switches: early modifications drive activation and assembly, intermediate ones optimize catalytic or transcriptional output, and late ones dismantle signaling modules to enforce irreversible shutdown. Sequential PTMs may add timing and directionality to molecular signals, thereby helping immune cells detect pathogens, control inflammation, and eventually return toward homeostasis.

### 5.2 Competitive PTMs as molecular decision points

Competitive post-translational modifications create mutually exclusive biochemical states by targeting the same amino acid residue or overlapping regulatory motifs. These modifications act as molecular decision points that direct distinct signaling pathways (204). Lysine residues exemplify this logic, as acetylation, ubiquitination, SUMOylation, methylation, and other acylations cannot coexist at a single site. The identity of the dominant PTM therefore influences whether a protein is stabilized, activated, repressed, or targeted for degradation. For instance, acetylation of NF-κB RelA/p65 enhances DNA-binding affinity and transcriptional persistence (205, 206), whereas ubiquitination at adjacent lysines promotes proteasomal turnover, rapidly terminating inflammatory signaling (207). Competitive interactions also occur in receptor-proximal pathways, where phosphorylation of IRAK-dependent adaptors promotes scaffold assembly and signal propagation (188). In contrast, ubiquitination of these regulatory domains opposes phosphorylation and initiates signal termination (201, 208). These mutually exclusive PTM states enable immune cells to rapidly and effectively shift regulatory functions without relying on new protein synthesis, allowing them to transition swiftly between activation, attenuation, and termination. Competitive PTMs may function as precise molecular switches that convert small signaling changes into distinct immune outcomes. This helps keep the immune system quick to react and well-controlled during fast-changing inflammation.

### 5.3 Hierarchical PTM relationships: overwriting and commitment

In addition to sequential and competitive interactions, PTMs may also exhibit hierarchical relationships in which certain modifications take precedence by reshaping protein structure, influencing complex formation, or initiating irreversible degradation. This hierarchy may reflect intrinsic biochemical relationships rather than substrate availability alone. Early PTMs can create permissive states that enable further regulation, whereas late PTMs may impose restrictive or irreversible outcomes that supersede earlier signals (209).

For example, phosphorylation often acts as an early signal by revealing binding spots, keeping interactions stable, or creating places where ubiquitin ligases can attach (210, 211). Once engaged, K48-linked ubiquitination overrides these activation signals by targeting substrates for proteasomal degradation (212). In contrast, non-degradative ubiquitin chains, SUMOylation, and acetylation can coexist with phosphorylation to fine-tune activity by modulating interaction networks, oligomerization, or nuclear trafficking (213, 214). Irreversible marks—such as degradative ubiquitination or SUMO-driven chromatin retention—may displace reversible ones like phosphorylation or acetylation, thereby enforcing pathway commitment.

Through such hierarchical relationships, PTMs may impose directional constraints that influence which regulatory signals persist and which are overwritten. Proteins first modified by activating marks can later acquire inhibitory or degradative post-translational modifications (PTMs) that reduce or end their function. In contrast, stabilizing PTMs may protect critical residues and extend protein activity beyond receptor engagement. This hierarchy suggests that, in some contexts, signaling transitions may proceed in a partially ordered manner, potentially contributing to activation, resolution, or more suppressive states.

### 5.4 Multi-PTM control of shared targets

Combinatorial PTM patterns represent a higher-order regulatory logic in which multiple modifications on the same substrate collectively encode protein function (68, 215, 216). Rather than acting as independent binary switches, co-existing PTMs form multi-layered biochemical states that influence protein stability, interaction networks, localization, and transcriptional potency (217, 218). Immune regulators therefore function as integrative hubs, with their activity shaped by the overall PTM composition and balance rather than any single modification. This architecture allows signaling molecules to integrate diverse upstream signals into unified outputs with a level of precision that individual PTMs cannot achieve. NF-κB RelA/p65 exemplifies this encoded regulation. Phosphorylation promotes nuclear entry and transcriptional activation (219), acetylation stabilizes DNA binding (206), whereas deacetylation limits transcriptional persistence (205, 220). K48-linked ubiquitination overrides both by directing RelA for proteasomal degradation (221). The resulting phenotype reflects the combined effects and quantitative balance of these modifications. Similar combinatorial logic governs histone and non-histone regulators involved in cytokine expression, inflammasome priming, and adaptor assembly (222–224), where clusters of PTMs sculpt multi-dimensional regulatory states that may exceed the influence of single marks.

Such integration allows immune pathways to rapidly reconfigure without requiring new transcription or protein synthesis. Instead of enforcing linear trajectories, combinatorial PTMs generate graded, modular regulatory states whose functional consequences depend on the collective PTM signature. To understand these PTM groups, we need tools that can identify specific sites where they occur and track how they change inside living cells. This is important because the effect of one PTM depends a lot on the other PTMs around it. Together, these principles position PTM assemblies as key configuration-dependent frameworks shaping immune behavior.

### 5.5 A proposed framework for PTM crosstalk and hierarchy in sepsis

Together, the principles of sequential ordering, competitive exclusion, and combinatorial encoding suggest that PTMs function not as isolated marks but as interconnected regulatory networks that shape immune signaling. PTM networks can layer reversible and irreversible modifications on shared substrates to build structures that can amplify, constrain, or redirect pathway outputs with context-dependent timing. Coordinated shifts in post-translational modifications, rather than changes in transcription or protein synthesis, may contribute to immune transitions between activation, attenuation, and suppression.

A key consequence of this organization may be the emergence of non-linear, switch-like immune transitions. Because multiple PTMs converge on the same regulatory nodes, small perturbations—such as displacement of one PTM by another or altered enzyme activity—can cross a threshold and rapidly reconfigure signaling outcomes. Such threshold dynamics may help explain how immune states in sepsis shift abruptly from hyperinflammation to tolerance (225).

Dysregulated PTM configurations may underlie the paradoxical coexistence of persistent upstream activation and downstream paralysis seen in sepsis (226). Stabilizing scaffold PTMs can maintain receptor-proximal signaling even as inhibitory or degradative modifications silence transcriptional regulators (73). Conversely, premature deposition of repressive PTMs can collapse signaling before effector functions are executed (188). These observations highlight the importance of PTM configurations as integrated regulatory structures, rather than individual modifications in isolation.

Overall, PTM networks may function as molecular switch-like regulators that influence the direction, persistence, and resolution of immune responses (227, 228). PTM networks store regulatory information using combinations, layers, and exclusive states (229). They may help explain how the immune system changes during sepsis. These networks also suggest potential ways to modulate the immune response by targeting PTM circuits instead of just single pathways (137, 146).

The context-associated patterns outlined in Section 4 do not operate independently; rather, they may reflect PTM interaction principles explained in Section 5. Sequential ordering, competitive modification, and hierarchical overwrite may together provide a conceptual framework through which PTMs may contribute to non-linear immune-state transitions in sepsis (230, 231).

The interconnected nature of PTM crosstalk challenges the effectiveness of targeting single enzymes. Instead, strategies that combine or sequence interventions to reshape PTM interactions may offer a more effective conceptual direction than simply eliminating individual modifications.

## 6 Controversies, knowledge gaps, and unresolved questions

While numerous studies are consistent with a PTM-associated framework of immune transition in sepsis, important conceptual, methodological, and clinical challenges persist. These gaps prompt important questions about whether PTMs actively determine immune outcomes, are secondary indicators of metabolic failure, or function within larger regulatory networks. Addressing these issues is essential for validating PTMs as actionable therapeutic targets rather than descriptive correlates.

### 6.1 Causality of lactylation in immunosuppression: active driver or epiphenomenon?

Histone lactylation, especially H3K18la, increases during late sepsis and has been associated with wound-healing or immunosuppressive macrophage programs. However, whether it is causally responsible for these effects remains unclear (127, 232, 233). Current evidence is largely correlative: lactylation increases alongside glycolytic overflow, mitochondrial dysfunction, and activation of wound-healing programs (234). This uncertainty is reinforced by recent studies showing that lactylation can also participate in trained-immunity–associated and innate memory-like programs, indicating that its functional consequences are likely to be context-dependent rather than restricted to late immunosuppression (116, 117). Because existing perturbations (LDHA inhibition, glycolytic blockade, hypoxia modulation) simultaneously alter multiple metabolic pathways, they cannot isolate lactylation from the broader metabolic collapse accompanying sepsis. Thus, it remains unclear whether lactylation drives immunosuppression or simply marks metabolically compromised cells.

A major barrier to establishing causality is the incomplete definition of the lactylation machinery. Although p300 can add lactyl marks *in vitro*, its physiological preference for lactyl-CoA over acetyl-CoA remains unclear (256, 257), and authentic lactyltransferases have yet to be identified *in vivo (*235, 236). In addition, no mammalian delactylase has yet been conclusively validated (226). The absence of validated delactylases limits the ability to selectively remove lactyl marks. This gap restricts the use of traditional loss-of-function methods to assess whether lactylation plays an important role in establishing or maintaining late immune tolerance.

At the same time, emerging studies suggest that lactylation may still be experimentally and therapeutically tractable even before its full enzymatic machinery is resolved. Recent work has shown that pharmacologic inhibition of lactate production or p300-associated histone lactylation can attenuate trained immunity phenotypes, whereas LDHA-dependent lactate metabolism contributes to both histone lactylation and secondary immune responsiveness (116, 117). These findings suggest that lactylation may be modulated at both metabolic and enzymatic levels, although the specificity, durability, and stage dependence of such interventions remain to be clarified.

Addressing these uncertainties requires targeted perturbations that influence lactylation without altering metabolic processes. This includes implementing lysine-to-arginine substitutions at critical histone sites, conducting biochemical analyses of potential lactylation writers and erasers, and developing probes capable of distinguishing lactylation from related acyl modifications. Time-resolved single-cell PTM profiling is critical to establish whether lactylation initiates transcriptional silencing or acts to reinforce an already repressed chromatin state.

Until tools capable of clarifying its role are developed, lactylation will remain an uncertain aspect of sepsis—either acting as a direct epigenetic driver of persistent immunosuppression or simply reflecting metabolic failure. Determining whether lactylation can be targeted for treatment or if it merely indicates advanced immune dysfunction is essential.

### 6.2 Causal direction: metabolic flux vs hierarchical signal ordering

A key question remains whether metabolic constraints directly trigger PTM shifts in sepsis or if upstream signaling pathways initiate these changes, with metabolism playing a secondary role. The Metabolic–PTM Temporal Switch model emphasizes acetyl-CoA, NAD^+^, and lactate availability as key factors that may influence context-associated post-translational modifications (PTMs) (237–239). In contrast, alternative models argue that PTM transitions result from predefined signaling pathways and transcription factor dynamics, with metabolic changes occurring subsequently (240). Distinguishing between these frameworks remains unresolved.

Evidence supporting a signaling-first model shows that chromatin repression, heterochromatin recruitment, and transcription factor silencing can occur before noticeable metabolic decline. This is especially evident in endotoxin tolerance, where regulatory complexes assemble despite glycolysis remaining intact (136, 241, 242). On the other hand, studies on macrophage activation show that metabolism happens first. Glycolysis speeds up quickly, ATP levels go up, and acetyl-CoA is made before changes in chromatin and protein modifications occur (243). These contradictory findings suggest that causal ordering may vary by cell type, stimulus strength, and timing of feedback circuits.

Spatial compartmentalization adds further complexity. PTMs at receptor complexes, inflammasomes, or endosomal scaffolds may depend more on local kinase–adaptor availability than on global ATP or acyl-CoA pools (244, 245). Mitochondrial ubiquitination and phosphorylation rely on organelle-confined substrates partially insulated from cytosolic metabolism (246, 247). Thus, metabolic gating may apply strongly to nuclear PTMs but less so to PTMs within signaling microdomains (248).

To resolve these competing hypotheses, it is necessary to apply targeted perturbations that independently adjust metabolic substrates and PTM machinery. This involves modifying metabolite pools while keeping PTM enzyme levels constant, conducting temporal single-cell PTM profiling to establish sequence, and using subcellular isotope tracing to evaluate local substrate utilization. These approaches may help determine whether metabolism acts as a trigger, a permissive background, or a reinforcing feedback loop in PTM-driven immune transitions.

Emerging evidence supports a bidirectional model where signaling directs the sequence and specificity of PTMs, while metabolic flux defines the biochemical boundaries that govern these processes (137, 239, 249). Defining their relative contributions remains a key frontier for understanding immune-state transitions during sepsis.

### 6.3 Context-dependent function: the bidirectional roles of PTM enzymes (e.g., SIRT1)

SIRT1 is widely viewed as a mediator of immune tolerance through NF-κB deacetylation, transcriptional repression, and chromatin condensation (250–252). Substantial evidence demonstrates that activating SIRT1 suppresses early hyperinflammation by limiting cytokine overproduction, reducing oxidative damage, and preserving mitochondrial function (253–255). These opposing roles reflect a key conceptual point: SIRT1 is not inherently immunosuppressive, but a bidirectional PTM regulator whose effects depend on metabolic state, timing, and cell identity (256).

A major source of ambiguity is SIRT1’s reliance on NAD^+^ (256, 257). Early in infection, when NAD^+^ levels and mitochondrial function remain intact, SIRT1 preferentially activates metabolic regulators such as AMPK and PGC-1α, restraining inflammation without inducing chromatin silencing (258–260). As sepsis advances and the NAD^+^/NADH ratio declines, the acetylation state may shift toward deacetylation-associated repression in a context-dependent manner, contributing to reduced antigen presentation and weakened cytokine responsiveness. In this setting, the relative contributions of SIRT1, other sirtuins, and classical HDACs likely vary by metabolic state, cell type, and subcellular context (28). Thus, SIRT1’s functional polarity may shift along the metabolic trajectory of sepsis (261).

These effects depend on the type of cell. In macrophages, SIRT1 promotes tolerance by suppressing NF-κB activity (251, 262, 263). In endothelial cells, it helps maintain barrier integrity (264–266). In T cells, it modulates active immune responses while preserving memory of past threats (267). In hepatocytes, it regulates metabolism and overall inflammation (268).

Global activation of SIRT1 can both protect organs and intensify immunosuppression, depending on which cellular compartments drive the response. This dual effect helps explain the inconsistent therapeutic outcomes observed in preclinical studies (269, 270).

Overall, SIRT1 illustrates a broader principle in PTM biology: acetylation/deacetylation enzymes rarely exert unidirectional immunologic effects (271). Their outcomes depend on the intersection of metabolic status, disease context, and cellular context (47). Effective SIRT1-targeted therapy will thus require context-specific and compartment-selective strategies, activating SIRT1 during early hyperinflammation but inhibiting it during late immunosuppression to restore NF-κB-dependent transcription (272). This highlights the need for models that combine metabolism, changes in protein modifications, and the specific details of each cell. Without these models, we might misunderstand how regulation works or miss chances for treatment (273).

### 6.4 Methodological imperatives: single-cell, spatial, and multi-PTM analysis

Defining the temporal logic of PTMs in sepsis is limited by major methodological constraints that obscure when, where, and in which cells specific modifications arise. Most studies rely on bulk proteomics or antibody-based assays (274), which lack the temporal and cell-type resolution needed to capture PTMs that turn over within seconds and vary markedly across immune compartments. Current measurements often capture only a single time point rather than dynamic trajectories, making it difficult to infer causality or reconstruct the order of metabolic, signaling, and chromatin events.

A central analytical gap is the inability to resolve co-occurring PTMs on the same peptide (36, 275, 276). Competitive marks (e.g., acetylation vs lactylation) or ordered modifications (e.g., phosphorylation to ubiquitination) are lost in bottom-up proteomics because peptide fragmentation destroys multi-PTM configurations (277)—yet these configurations likely encode the true regulatory “code.” Advanced tools such as top-down proteomics and multi-PTM spectral deconvolution offer promise but currently lack sensitivity, throughput, and robustness for clinical or longitudinal use (278).

Integration of metabolic flux with PTM profiling is similarly poor (279, 280). Conventional proteomics rarely incorporates stable isotope tracing, making it difficult to distinguish substrate-driven PTMs from signaling-driven ones (281, 282). Spatial context is also underexplored: lactylation detected in nucleus, cytosol, and mitochondria likely reflects distinct processes, yet these compartments are typically analyzed together (106, 283, 284). Approaches such as organelle-resolved proteomics, isotope-labeled acyl donors, and PTM-resolved chromatin profiling (e.g., CUT&RUN/CUT&Tag for acyl marks) will be required to disentangle these layers (285).

Sampling limitations create additional barriers. Most human studies look at circulating PBMCs, even though important immune changes happen in cells that stay in tissues—like lung macrophages, liver Kupffer cells, spleen APCs, and bone marrow progenitors. These PTM programs cannot be fully inferred from blood alone. Without cell- and tissue-resolved mapping, systems-level models of PTM regulation remain incomplete (286).

Finally, clinical translation is hampered by the lack of scalable, real-time PTM assays. Even when high-resolution proteomics is feasible, sample volume, cost, and slow turnaround preclude serial monitoring in critically ill patients. Progress will require minimally invasive technologies such as PTM-selective liquid biopsies, nanoengineered capture probes, or imaging-compatible PTM sensors.

Collectively, these limitations impede reconstruction of PTM hierarchies, causal relationships, and mechanistic drivers. Overcoming them will require advances in single-cell proteomics, multi-PTM co-detection, metabolic tracing, and clinically deployable monitoring platforms.

### 6.5 Translational gaps: longitudinal human data and context-informed intervention

Despite advances in characterizing PTM-mediated immune regulation, most mechanistic insights come from short-term animal models or acute *in vitro* systems that mainly capture early hyperinflammation (132, 287, 288). By contrast, patient-derived sepsis datasets and clinical observations mainly provide disease relevance and longitudinal context, but usually lack the temporal and mechanistic resolution needed to reconstruct PTM order directly. These models rarely progress into the metabolic collapse and chromatin remodeling that define late immunosuppression (289), leaving the full temporal organization of PTM transitions largely inferred rather than empirically mapped. The common use of single timepoint sampling—often within hours of LPS exposure—further prevents reconstruction of PTM kinetics and differentiation between transient signaling events and context-defining PTM shifts (76).

Human studies introduce additional challenges. Nearly all rely on peripheral blood mononuclear cells (290–292), even though key immunologic reprogramming in sepsis (macrophage tolerance (293), neutrophil dysfunction (294), antigen-presentation failure (295)) occurs within tissue-resident compartments of the lung, liver, spleen, and bone marrow. These niches have distinct cytokine gradients, hypoxia, and metabolic profiles (296), meaning circulating PTM signatures may reflect diluted or indirect surrogates of organ-level processes. Without spatial sampling, organ-specific PTM trajectories remain inaccessible.

Clinical heterogeneity—differences in pathogen type, infection site, comorbidities, treatment, and timing—further complicates interpretation (297–299). Cross-sectional datasets that mix early and late samples risk conflating hyperinflammatory and immunosuppressed states (300, 301). Prospective longitudinal cohorts with serial sampling over days to weeks are essential to infer directional PTM changes and define clinically meaningful disease contexts (302–304).

New technologies offer possible solutions. These include single-cell proteomics (305–307), low-input MS (308, 309), microvolume sampling over time (310, 311), and time-based metabolomics. These approaches may help track changes in protein modifications along with metabolism and immune system activity (312). Coupling these tools with modern sepsis genotyping (immune subtypes (313), SOFA trajectories (301, 314, 315), lactate kinetics (316)) could allow PTM-associated patient stratification and identification of context-informed therapeutic windows.

Overall, the lack of longitudinal, tissue-resolved, and clinically stratified datasets remains a major barrier to validating PTMs as causal drivers rather than passive correlates of severity. Closing this gap is critical for translating PTM hierarchies into actionable biomarkers and therapeutic strategies.

### 6.6 Challenges in translating PTM modulation into therapy

Translating PTM-based regulatory insights into therapy remains challenging because PTM enzymes function within interconnected networks linking transcription, metabolism, and proteostasis (317, 318). Pharmacological modulation of core regulators such as p300/CBP, SIRT deacetylases, and E3 ligases can have widespread effects. Since these molecules play critical roles in maintaining homeostasis (319–321), targeting them raises concerns about widespread toxicity and unintended immunosuppression (318, 322, 323). Effective intervention therefore requires precision modulation of PTM states, not global inhibition or activation of PTM enzymes (324).

A major barrier is the extreme pleiotropy of PTM writers and erasers (185, 325, 326). Enzymes like SIRT1 and p300 modify hundreds of substrates across diverse lineages, influencing mitochondrial function, DNA repair, and transcription (327). Efforts to target specific modifications, such as H3K18 lactylation or RelA/p65 acetylation, face challenges due to the lack of residue-specific tools and incomplete identification of delactylases and PTM-specific cofactors (328, 329). These limitations hinder the ability to suppress pathogenic PTMs without affecting essential cellular functions (330, 331).

For lactylation specifically, the most plausible intervention routes currently include modulation of lactate generation or utilization, inhibition of LDHA-dependent metabolic flux, and interference with p300-associated lactylation pathways. Recent studies showing that LDH or p300-related perturbation can reduce trained-immunity phenotypes provide proof of principle that lactylation-linked immune states may be pharmacologically modifiable (116, 117). However, because these interventions affect broader metabolic and chromatin programs, their effects cannot yet be interpreted as lactylation-specific, underscoring the need for more selective writer/eraser tools and residue-resolved perturbation systems.

Spatiotemporal heterogeneity further complicates therapy (332, 333). PTM patterns are different depending on the tissue, cell type, and subcellular compartment (334–336). Suppressing macrophage hyperactivation with a widespread agent can also disrupt dendritic cell priming, T-cell metabolism, or the stability of the endothelial barrier. Achieving selectivity will require organ-targeted carriers (337), immune-cell–specific delivery (324, 338, 339), or compartment-directed therapeutics such as mitochondria-targeted PTM modulators (340, 341).

Timing is equally critical. Because the relative prominence of PTM-associated patterns may shift across sepsis-associated immune states (187, 342), the same drug can have opposite effects depending on immune context (343, 344). Early inhibition of acetyltransferases might blunt cytokine storm but accelerate chromatin silencing (345, 346); late SIRT1 inhibition may restore antigen presentation yet worsen early inflammation if mis-timed (347, 348). Thus, therapy must be aligned with real-time PTM biomarkers rather than fixed schedules (349, 350).

PTM-targeted interventions are also constrained by metabolic state (351). Enzymatic activity depends on NAD^+^, acetyl-CoA, and lactyl-CoA (352–354); severe mitochondrial or redox failure may render PTM-directed drugs ineffective unless metabolic deficits are corrected (355). Supporting mitochondrial function and restoring redox balance may be prerequisites for reclaiming PTM regulatory capacity (356).

These challenges emphasize the need to approach PTM therapy as a reprogramming of PTM-associated immune states. This approach should combine PTM modulators with metabolic support, target delivery to specific organs, and employ energetic diagnostics to determine a patient’s PTM-associated immune state. This reframes treatment as restoring adaptive immune transitions rather than merely blocking or stimulating isolated inflammatory pathways.

Addressing these gaps will require technological advances that resolve temporal, spatial, and combinatorial PTM complexity. Multi-PTM codetection tools—including enhanced top-down proteomics, multi-PTM spectral deconvolution, and hybrid enrichment—play a critical role in decoding sequential and competitive modifications. Also, stable isotope tracing links metabolic processes to PTMs, while PTM–chromatin integrated assays such as acyl-optimized CUT&RUN and CUT&Tag provide comprehensive insights into these interactions. These next-generation methods may help reconstruct causal PTM hierarchies *in vivo* and reveal how PTM-associated immune states develop and stabilize during sepsis.

These uncertainties emphasize the need for intervention strategies that adjust in real time, guided by biomarkers to actively regulate PTMs as immune and metabolic conditions evolve during sepsis progression.

## 7 Translational perspectives: targeting immune signaling across PTM-associated immune states

**Figure 4:**
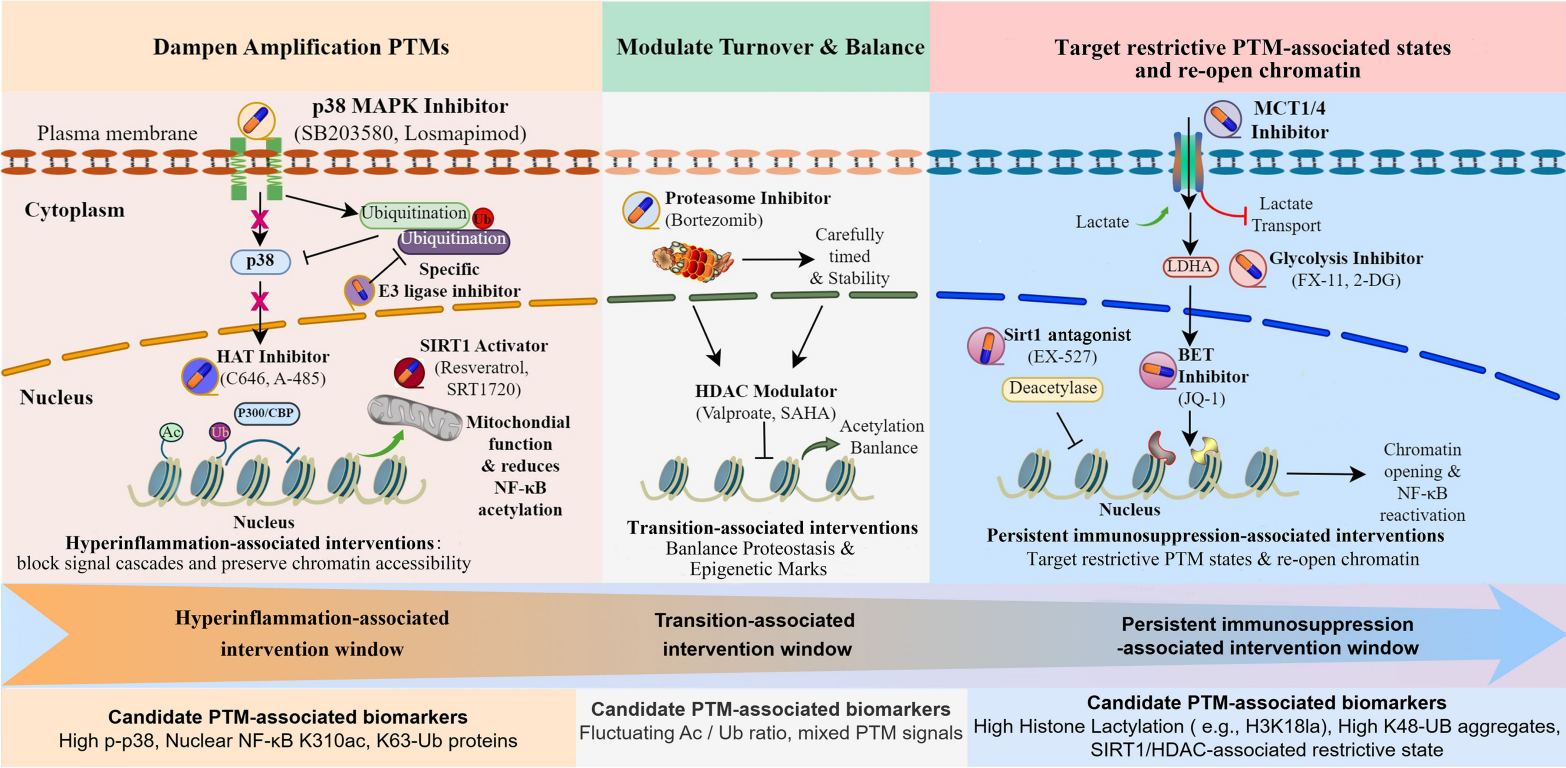
Conceptual framework for context-informed immune intervention across PTM-associated immune states in sepsis. This figure is intended as a conceptual and context-informed framework and does not imply a fixed or universally conserved temporal sequence *in vivo*. This diagram outlines a therapeutic framework based on the Metabolic–PTM Temporal Switch model. In hyperinflammation-associated contexts, interventions may aim to restrain excessive signaling by targeting kinases, histone acetyltransferases, or selected E3 ligases, thereby reducing cytokine release while preserving core signaling competence. In transition-associated contexts, therapeutic strategies may focus on limiting proteostatic collapse and maintaining transcriptional responsiveness; this includes timed proteasome modulation or HDAC-directed intervention. In persistent immunosuppression-associated contexts, therapeutic strategies may need to reverse metabolic and epigenetic constraints, including modulation of lactate production or transport, rebalancing NAD^+^–dependent deacetylation pathways, reducing lactylation-associated programs, and reopening restrictive chromatin states. The same molecular target may exert different effects depending on timing and immune context. Together, these considerations highlight the need for PTM-informed stratification and biomarker-guided treatment strategies in sepsis.

A central challenge in sepsis therapy is the repeated failure of immune modulation strategies that apply uniform interventions across biologically distinct immune contexts (357). Clinical trials targeting cytokines (358), checkpoint pathways (359), or immune stimulation have often produced inconsistent or paradoxical outcomes (360), suggesting that immune dysfunction in sepsis cannot be corrected by altering inflammatory intensity alone (361). Therapeutic success may depend on restoring signaling competence within a tightly regulated time frame, requiring immunomodulatory strategies to be aligned with predominant PTM–metabolic constraints rather than uniformly applied across all patients or disease contexts (**Figure 4**).

Building on the Metabolic–PTM Temporal Switch model outlined in this review, sepsis can be understood as involving PTM-associated signaling states that may present distinct molecular vulnerabilities and therapeutic opportunities. From this perspective, effective intervention may require alignment with the predominant PTM-associated constraints shaping immune behavior in a given context, rather than indiscriminate immune activation or suppression.

### 7.1 PTM-associated signaling states as potential biomarkers

Traditional biomarkers in sepsis—such as circulating cytokines (362–364), lactate levels (365, 366), or leukocyte counts—reflect disease severity but provide limited insight into the functional state of immune signaling networks (367). In contrast, PTM signatures may more directly reflect signaling capacity and pathway integrity, making them attractive candidates for context-informed biomarkers.

Hyperinflammation-associated states may feature strong phosphorylation of MAPKs and TBK1, accompanied by K63-linked ubiquitination that forms scaffolds on receptor-proximal complexes and inflammasomes (368). More restrictive transition-associated states may show increased K48-linked ubiquitination and accelerated proteasomal degradation of signaling adaptors. They may also feature more prominent deacetylation alongside declining HLA-DR expression (369), indicating a loss of transcriptional competence (370, 371). More persistent immunosuppression-associated states may be characterized by metabolic lactate accumulation (372, 373), sustained histone lactylation (notably H3K18la), and stable chromatin repression at inflammatory loci.

**Figure 5:**
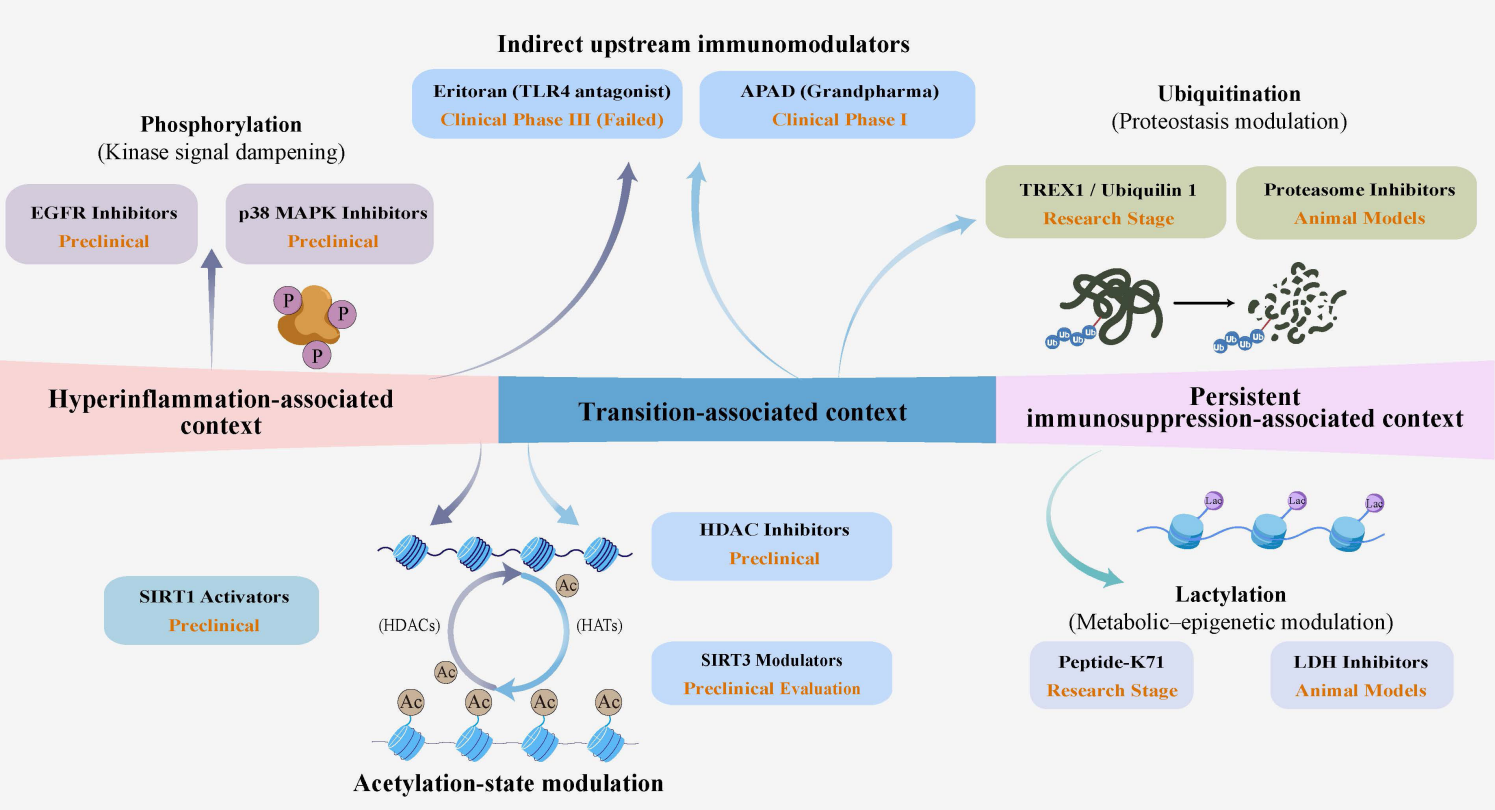
Context-associated landscape of PTM-targeted therapeutic development in sepsis. Schematic overview of pharmacological strategies that intersect with post-translational modification (PTM) networks across representative sepsis-associated immune contexts, including hyperinflammation-associated, transition-associated contexts, and persistent immunosuppression-associated contexts. Interventions are grouped by dominant PTM layers and shared mechanistic logic (e.g., kinase signal dampening, proteostasis modulation, and metabolic–epigenetic modulation) rather than by individual agents. Upstream immunomodulators (e.g., TLR4 antagonists) are displayed separately, as they may reshape downstream PTM landscapes by attenuating proximal inflammatory inputs rather than directly targeting PTM enzymes. Developmental status is annotated to indicate translational maturity (e.g., research stage, preclinical, animal models, clinical evaluation, or failed trials) and to highlight context-dependent feasibility and limitations. This figure is intended as a conceptual and context-informed overview and does not imply a fixed or universally conserved temporal sequence *in vivo*.

Integrating PTM profiling—via phosphoproteomics, ubiquitin linkage–specific assays, or targeted chromatin PTM detection—into clinical stratification could enable real-time mapping of immune trajectory. PTM-informed stratification may allow therapeutic decisions to be based on signaling state rather than relying solely on static inflammatory markers. A conceptual overview of PTM-targeted therapeutic development and clinical maturity is shown in **Figure 5**, and mechanistic details, outcomes, and key limitations are summarized in **Table 1**.

**Table 1:** Representative PTM-associated therapeutic strategies in sepsis: immune context, mechanisms, and limitations.

Regulatory layer/intervention class	Representative target/intervention	Representative immune context	Proposed/reported mechanism and representative outcome	Key limitations & risks
Phosphorylation	EGFR Inhibitors	Hyperinflammation-associated context	Promotes M1 to M2 macrophage transition; alleviates sepsis-induced ALI.	EGFR kinase activity is crucial for receptor trafficking and activation.
	p38 MAPK inhibitors (e.g., SB203580)	Hyperinflammation-associated context	Reduces cytokine storm by blocking rapid signal amplification.	Prolonged use may impair host defense and pathogen clearance.
Ubiquitination	Proteasome Inhibitors (e.g., Bortezomib)	Transition-associated context	Transiently preserves signaling competence by preventing scaffold degradation.	High systemic toxicity; non-selective disruption of proteostasis.
	TREX1/Ubiquilin 1	Transition-associated context	Mono-ubiquitination regulates TREX1 ER localization and immune signaling.	Mechanisms regarding ER-associated degradation are still being explored.
Acetylation-state modulation	HDAC Inhibitors (e.g., Valproate, SAHA)	Hyperinflammation/transition-associated contexts	Attenuates excessive inflammation via epigenetic tuning.	Risk of accelerating immune tolerance if administered during persistent immunosuppression-associated contexts.
	SIRT3 Modulators (e.g., Emodin)	Hyperinflammation/transition-associated contexts	Regulates cardiac TCA cycle enzymes to treat sepsis-induced myocardial dysfunction (SIMD).	Currently limited to organ-specific protective assessments.
	SIRT1 Activators (e.g., Resveratrol)	Hyperinflammation-associated context	Provides organ protection and maintains mitochondrial function.	May worsen immunosuppression if used in persistent immunosuppression-associated contexts.
Lactylation	LDH Inhibitors (e.g., FX-11, 2-DG)	Persistent immunosuppression-associated context	Partially restores immune responsiveness by reducing lactylation.	Potential metabolic toxicity and indirect regulatory effects.
	Peptide-K71 (Targeting ENO1-K71)	Persistent immunosuppression-associated context	Inhibits ENO1 lactylation to prevent microvascular leakage and dysfunction.	Drug-like properties of specific peptides require further validation.
General Immuno-modulators	APAD (Grandpharma)	Hyperinflammation/transition-associated contexts	Assesses safety and PK in healthy volunteers; antagonizes PAMPs.	Clinical efficacy in septic patients yet to be determined in Phase II/III.
	Eritoran (TLR4 Antagonist)	Hyperinflammation-associated context	Attempted to reduce 28-day mortality by blocking TLR4/CD14.	Failed to show mortality benefit compared to placebo.

A translational implication of this framework is that PTM-informed stratification may need to distinguish infection-associated sepsis from sterile critical illness states such as major trauma or burns, even when overlapping metabolic and epigenetic signatures are present, because the balance between PAMP- and DAMP-driven reprogramming may influence both biomarker interpretation and treatment timing.

### 7.2 Hyperinflammation-associated intervention window

In hyperinflammation-associated contexts, immune signaling retains high plasticity and reversibility (374, 375). Phosphorylation-driven kinase cascades and non-degradative ubiquitination amplify innate immune responses efficiently (121, 151), but excessive persistence of these signals contributes to tissue damage and organ failure (376, 377).

In this context, therapeutic strategies could aim to restrain signal amplification while preserving downstream signaling architecture. Selective modulation of kinase activity, inflammasome priming, or scaffold-forming ubiquitination may reduce cytokine excess without precipitating premature signal collapse. Importantly, interventions that directly dismantle signaling complexes or enforce chromatin silencing in this context risk accelerating transition toward immune incompetence (288).

Thus, this context may represent a window for signal tuning, not signal termination.

### 7.3 Transition-associated intervention window

The transition-associated context may represent a critical commitment point in which immune fate becomes directionally constrained (378–380). Ubiquitin linkage editing—from K63-linked scaffolding to K48-linked degradation—combined with more prominent deacetylation may help dismantle signaling complexes and narrow transcriptional accessibility. Once this threshold is crossed, immune reactivation becomes increasingly difficult (381).

Therapeutic opportunities in this context center on preserving or restoring signaling competence. Targeting specific E3 ligases or deubiquitinases that enforce degradative ubiquitination may stabilize receptor-proximal signaling modules (382–384). Concurrently, selective modulation of HDACs or NAD^+^-dependent deacetylases may reopen transcriptional windows without fully reinitiating hyperinflammation (385, 386).

Because interventions in this context can either restore competence or potentially accelerate immune silencing, timing and patient stratification are critical. PTM-informed diagnostics may play an essential role in identifying individuals who remain within this reversible window.

### 7.4 Persistent immunosuppression-associated intervention window

Persistent immunosuppression-associated states may be characterized by structural loss of signaling capacity (387). Proteostatic dismantling of key adaptors, persistent chromatin condensation, and accumulation of long-lived PTMs—such as histone lactylation—may stabilize a low-plasticity immune state refractory to conventional immunotherapies (103, 105). In this context, strategies that rely solely on receptor agonism or cytokine supplementation are unlikely to succeed because the intracellular mechanisms needed to process these signals have been impaired. Therapeutic approaches must instead target upstream constraints, including metabolic exhaustion and epigenetic constraints.

Modulating the lactate–lactylation axis, restoring mitochondrial function, or reopening chromatin accessibility may be prerequisites for re-establishing signaling competence. However, given the persistence and limited reversibility of these PTM-associated states, such interventions may require combination strategies and prolonged reprogramming rather than acute stimulation.

### 7.5 A timing-centric framework for targeted therapy

These factors emphasize a key principle: the success of immune-directed therapy in sepsis may depend more on the timing of intervention in relation to PTM-associated signaling states than on the specific target chosen. The same molecular target—such as a kinase, deacetylase, or ubiquitin ligase—may exert protective or deleterious effects depending on when it is modulated along the disease trajectory.

By positioning PTMs as both biomarkers and mechanistic regulators of immune signaling competence, the Metabolic–PTM Temporal Switch model may provide a framework for precision immunotherapy in sepsis. Future strategies should move beyond simply suppressing or activating immunity. Instead, they should aim to guide patients through adaptive signaling changes by reducing amplification in more inflammatory contexts, preserving functional capacity in transition–associated states, and restoring signaling pathways in more persistent immunosuppression-associated states.

### 7.6 Outlook: from pathway targeting to signaling state reprogramming

Moving forward, translation of PTM biology into clinical practice will require technologies capable of resolving signaling states in real time, including low-input proteomics, PTM-specific liquid biopsies, and integrative metabolic–epigenetic profiling. Redefining sepsis therapy as a process of signaling state reprogramming, rather than simple pathway inhibition, may help explain why past clinical approaches have fallen short and may also highlight new opportunities for targeted, context-informed treatments.

## 8 Conclusion and perspectives

Sepsis has long been understood as a shifting imbalance between hyperinflammation and immunosuppression; however, this view does not fully account for the persistent immune dysfunction that resists therapeutic treatment. By framing sepsis as a context-dependent and multi-layered process of immune reprogramming, this framework may help explain how immune signaling gradually loses its adaptability and becomes resistant to treatment.

This model integrates metabolic gating, chromatin accessibility, and post-translational modification-associated regulation to interpret immune activation, resolution, and suppression along a common regulatory framework. Rather than proposing a fixed or universally conserved sequence of PTM dominance, it highlights how representative PTM-associated configurations, including phosphorylation, ubiquitination, acetylation/deacetylation, lactylation, and more durable epigenetic programs such as DNA methylation, may emerge under different metabolic and chromatin conditions. In this framework, immune failure in late sepsis may arise from impaired signaling competence and transcription constraint, rather than simply from insufficient inflammation.

Importantly, the current framework should be interpreted primarily in the context of myeloid immune dysfunction and tolerance-associated reprogramming, rather than as a universally conserved PTM sequence across all cellular compartments in sepsis.

From a translational perspective, these insights argue against one-size-fits-all immunomodulation and instead support context-informed, biomarker-guided strategies targeting PTM-associated immune states. By intervening at context-appropriate points of immune-state transition, it may become possible to restrain pathological inflammation without precipitating collapse, or to stabilize immune function without re-igniting cytokine storm. Overall, focusing on PTM-associated immune states may provide a useful conceptual framework for addressing the persistent therapeutic challenges of sepsis.
